# Impact of COVID-19 and intensive care unit capacity on vaccination support: Evidence from a two-leg representative survey in the United Kingdom

**DOI:** 10.1016/j.jve.2021.100044

**Published:** 2021-05-17

**Authors:** Géraldine Blanchard-Rohner, Bruno Caprettini, Dominic Rohner, Hans-Joachim Voth

**Affiliations:** a Pediatric Immunology and Vaccinology Unit, Geneva University Hospitals and Faculty of Medicine, Geneva, Switzerland; b Center of Vaccinology, Geneva University Hospitals, Switzerland; c Department of Economics, University of Zurich, Switzerland; d Department of Economics, University of Lausanne, Switzerland

**Keywords:** Vaccine, Vaccination hesitancy, Vaccine scepticism, COVID-19, Intensive care unit (ICU) capacity, Trust in medical experts

## Abstract

**Background:**

Overcoming coronavirus disease (COVID-19) will likely require mass vaccination. With vaccination scepticism rising in many countries, assessing the willingness to vaccinate against COVID-19 is of crucial global health importance.

**Objective:**

The goal of this study was to examine how personal and family COVID-19 risk and ICU (intensive care unit) availability just before the pandemics influence the acceptance of future COVID-19 vaccines.

**Methods:**

A two-leg survey was carried out for comparing vaccination attitudes pre-and post-COVID-19. UK residents were surveyed in October 2019 about their vaccination attitudes, and again in a follow-up survey in April 2020, containing the previous questions and further ones related to COVID-19 exposure and COVID-19 vaccine attitudes. The study combined survey results with local COVID-19 incidence and pre-COVID-19 measures of ICU capacity and occupancy. Regression analysis of the impact of individual and public health factors on attitudes towards COVID-19 vaccination was performed.

**Results:**

The October 2019 survey included a nationally representative sample of 1653 UK residents. All of them were invited for the follow-up survey in April 2020, and 1194 (72%) participated. The April 2020 sample remained nationally representative. Overall, 85% of respondents (and 55% of vaccine sceptics) would be willing to be vaccinated against COVID-19. Higher personal and family risk for COVID-19 was associated with stronger COVID-19 vaccination willingness, whereas low pre-COVID-19 ICU availability was associated with lower trust in medical experts and lower COVID-19 vaccine support. Further, general vaccination support has risen during the COVID-19 pandemic.

**Conclusion:**

Support for COVID-19 vaccination is high amongst all groups, even vaccine sceptics, boding well for future vaccination take-up rates. Vaccination willingness is correlated with health care availability during the COVID-19 crisis, suggesting a powerful synergy between health care system performance during crisis and the general population's trust in the medical profession – as reflected in vaccination support.

## Introduction

Since January 2020, SARS-Cov-2 has infected over 50 million people worlwide, leading to more than 3.2 million deaths. As of November 2020, there was no effective treatment; only large-scale vaccination will allow a return to ‘normal life’. The novel threat posed by COVID-19 and need for mass vaccination comes at a time of growing vaccine hesitancy – a reluctance or refusal to vaccinate despite vaccine availability – in developed countries.^1^ For example, only 47% of French citizens, 67% of Germans, and 72% of Americans currently believe that vaccines are safe.^2^ Accordingly, vaccination rates have fallen in many rich countries in recent years, such as in the United Kingdom (UK), France and the Netherlands.^3^^,^^4^ Even before the COVID-19 crisis, the World Health Organization ranked vaccine hesitancy as one of the top ten global health threats.^5^ For all these reasons, the National Institutes of Health (NIH) recently expressed concern that vaccine scepticism could undermine COVID immunization efforts.^6^

Vaccine hesitancy creates a major risk of suboptimal health outcomes for the general population^7^ and has several causes^8^^,^^9^: lack of confidence, complacency, constraints, calculation, and lack of collective responsibility. Growing vaccination fatigue and dropping vaccination rates have variously been attributed to low public trust in vaccines and health system, lack of perceived risks, wrong beliefs and misinformation, including the spread of fake news through social media, constraints on affordable and accessible vaccines, and insufficient governmental vaccine investments.^5^^,^^10^, ^11^, ^12^, ^13^, ^14^ Significant resistance to the measles vaccine can be traced back to a single scientific paper retracted in 2010.^15^

Further, the limited risk of contracting vaccine-preventable diseases (VPDs) and perceived low severity of VPDs have arguably reduced vaccination rates prior to COVID-19.^11^ Also, “free-riding” is a distinct possibility, with citizens deliberately benefitting from the vaccination efforts of others while reducing their own, resulting in socially sub-optimal vaccination rates.^7^

In general, few socio-economic variables predict vaccine scepticism. While education and higher income are associated in some countries with higher vaccination rates, in others they are correlated with vaccine hesitancy.^16^ Older respondents are generally less vaccine-hesitant, while the unemployed tend to be more skeptical.^17^ Individual vaccination choices are often strongly influenced by a person's social network and prevailing local social norms.^16^ In a representative sample of UK residents, almost a third of respondents were vaccine-hesitant for at least half (5 out of 10) of the assessed questions.^17^ Lack of awareness of vaccines' benefits and fear of side-effects were the main reasons for vaccine-scepticism. In particular, 79% of vaccine-hesitant respondents stated a lack of trust in immunization programs as the reason for hesitancy.^12^

To date, few interventions have been successful in overcoming vaccine hesitancy. While information provision can have some effect (such as e.g. educational pamphlets or web-based decision aids), many interventions yield neutral or counterproductive results.^18^ In particular, only half (5 out of 10) of the interventions using educational information to boost vaccination rates have led to a significant increase in vaccination intentions.^13^ Even in the case of successful interventions,^19^ a substantial share of anti-vaccine militants remains who are concerned about autism and bowel disease, despite being confronted with robust scientific evidence. Proximity to actual disease outbreaks – as in the case of measles – appears to have no overall effect on attitudes towards vaccines.^20^

The historic COVID-19 pandemic may affect attitudes towards vaccination for several reasons: First, COVID-19 involves higher personal stakes than many other diseases. In particular, some vaccine-preventable diseases are perceived as relatively mild and have a low probability of infection.^11^ In contrast, because COVID-19 is highly infectious and has a relatively high case-mortality rate with no hope for herd immunity anytime soon, incentives for vaccination are greater. Second, suspension of social distancing measures and economic recovery will require successful immunization of large portions of the global population against COVID-19.^21^ Third, the success of public health systems in dealing with the historic COVID-19 challenge may affect support for alternative (non-scientifically evaluated) approaches and methods.

## Methods

Our study aims to assess attitudes towards COVID-19 vaccination and to examine how the COVID-19 crisis affects support for vaccination in general. The effect of socio-economic characteristics and public health parameters on these attitudes were also considered. Our data comes from two surveys and a number of publicly available sources.

First, we surveyed a nationally representative sample of 1653 UK residents in October 2019 (pre-COVID-19) about their vaccination attitudes. The survey was administered by YouGov on the internet and incentivized participation with small lottery prizes. Respondents were sampled from a pool of individuals registered on the YouGov platform and willing to participate in surveys. For the sake of ensuring a representative sample, they were selected with a quota sampling method, using stratification by age, gender and education (joint), social grade, political attention, 2017 vote and region (joint) and 2019 EU vote and region (joint). Quota targets were taken from the most recent official statistics (census and electoral data). Surveys are a powerful tool for predicting actual vaccination decisions.^22^

This first survey mainly asked whether respondents would favor penalties for parents who refuse to vaccinate their children, in the form of fines, child benefit withdrawal, or bans from school. The same 1653 UK residents were contacted again for a second survey in April 2020 at the peak of the COVID-19 pandemic. For these individuals, information on pre-COVID-19 (October 2019) values of vaccination attitudes and socio-economic characteristics (age, gender, education level, income class, political attitudes) as well as the respondent's approximate location (first four digits of the UK postcode) were available. In addition, the April 2020 follow-up survey asked a set of additional questions about the perceived risk of COVID-19 for respondents or their immediate family, attitudes towards COVID-19 vaccination, and a battery of questions on political views. The survey questions are listed in the Supplementary Material.

We combined these survey results with a series of indicators of disease incidence and health care availability for the public health care system (NHS) in the area of residence. In particular, we used data from the Office of National Statistics^23^ on the number of deaths from COVID-19 occurring until 10^th^ April 2020, four days before the start of the survey (14^th^ April 2020) at the local authority level. The study has also examined how intensive care unit (ICU) capacities in late February 2020 affected vaccination attitudes. The ICU availability could reflect deep-seated, long-run factors that simultaneously determine the allocation of public healthcare infrastructure and vaccination attitudes, instead of capturing health care performance during the COVID-19 crisis. To rule this out, we used past ICU availability (i.e. from late February 2020).

We used Ordinary Least Squares (OLS) multivariate regressions to assess the relative importance of factors determining attitudes towards COVID-19 vaccination (specifications with probit estimators are shown in Tables S2 and S5). Our main dependent variable was an indicator variable taking value 1 if the respondent stated that he definitely or probably would not be vaccinated against COVID-19. The specification controlled for numerous potential confounders, including pre-COVID-19 attitudes towards vaccines in general (the questions asked in October 2019), the local COVID-19 incidence (COVID-19 deaths per 1000 people), all individual socio-demographic characteristics available (gender, 3 age categories, two social class indicators and 3 education level indicators), as well as for local population characteristics (share of population above 65 years old and life expectancy at 65 years old for men and women separately) which were selected to control for potential vulnerability of different areas of England. Standard errors were clustered at the level of the local authority to account for intra-cluster correlation of errors. We reported results for the full sample and three subsamples which were labelled as “no vax,” “hesitants,” and “pro vac.” Respondents were assigned to one of these three categories based on their answers to a set of 8 questions. Each question presented a statement about vaccines and asked the respondent to rate it on a 4-level scale: “definitely true,” “probably true,” “probably false” and “definitely false.” We generated an aggregate score based on these answers and used it to divide respondents into the three categories (See Section S.2 in the Supplementary Materials for additional details).

All participants have given written consent for survey participation and results to be published. All the statistical analysis and data storing used anonymized data that did not allow to identify individual participants.

The current study has been approved by the institutional ethics review board of HEC Lausanne, University of Lausanne (assigned acronym: VACAT). This study has been recorded at ClinicalTrials.gov, with identifier NCT04352582.

## Results

1653 (i.e. 93% of the respondents registered for participation and directed to the specific survey on vaccine attitudes) completed the questionnaire. 1194 (72%) of the initial participants responded to the second survey. This follow-up survey in April 2020 was also nationally representative (see Table 1). Average socio-demographic characteristics were very similar for the October 2019 and April 2020 samples and the average of the UK population as a whole. When studying if selection bias could be a concern, the non-response rates were not correlated with answers to the October 2019 vaccination questions (Table S1).

**Table 1:** Characteristics of survey respondents and representativeness of samples.

	United Kingdom		Survey respondents		Difference 2019–2020	p-value
			2019	2020		
Totals	61′371′315		1′653	1′194		

North East^a^	2′596′886	4%	5%	4%	0.55%	0.4781
North West^a^	7′052′177	11%	10%	10%	0.56%	0.6243
Yorkshire and the Humber^a^	5′283′733	9%	9%	8%	0.72%	0.5001
East Midlands^a^	4′533′222	7%	9%	10%	−0.97%	0.3796
West Midlands^a^	5′601′847	9%	8%	8%	0.34%	0.7396
East of England^a^	5′846′965	10%	12%	12%	−0.74%	0.5481
London^a^	8′173′941	13%	11%	10%	0.60%	0.6067
South East^a^	8′634′750	14%	12%	13%	−0.69%	0.5878
South West^a^	5′288′935	9%	10%	11%	−0.81%	0.4878
Wales^a^	3′063′456	5%	6%	5%	0.40%	0.6427
Scotland^a^	5′295′403	9%	9%	9%	0.04%	0.9715

Men^a^	30′140′820	49%	44%	46%	−1.64%	0.3849
Women^a^	31′230′495	51%	56%	54%	1.64%	0.3849

18-34 y.o.^a^	13′961′474	29%	25%	20%	5.00%	0.0016
35-54 y.o.^a^	17′054′980	35%	33%	34%	−0.59%	0.7411
55+ y.o.^a^	17′341′897	36%	42%	47%	−4.41%	0.0194

High social class^b^^,^^c^	21′381′588	57%	59%	59%	0.01%	0.9945
Low social class^b^^,^^c^	16′389′669	43%	41%	41%	−0.01%	0.9945

Education: entry level^b^	14′701′183	31%	29%	30%	−0.82%	0.6441
Education: some qualification^b^	9′548′605	27%	30%	31%	−0.59%	0.7419
Education: university^b^	11′059′503	42%	41%	40%	1.41%	0.4619

*Notes*: Col. 1 and 2 report totals and shares for the United Kingdom. Col. 3 reports characteristics of respondents to the 6–7 October 2019 survey. Col. 4 the characteristics of respondents on 9–16 April 2020. Every respondent in April 2020 completed the previous survey. Col. 5 reports the difference between col. 3 and col. 4. Col. 6 reports the *p-*value of a test that this number is different from 0, showing absence of differential attrition for all but 1 variable (age).

a 2011 Population Census.

b 2014 Integrated Household Survey; total numbers represent sum of weights.

c Social class is National Statistics Socio-economic Classification for the United kingdom and NRS social grade for the samples. High social class is 1–4 in NSSEC and A-C1 in NRS.

**Fig. 1:**
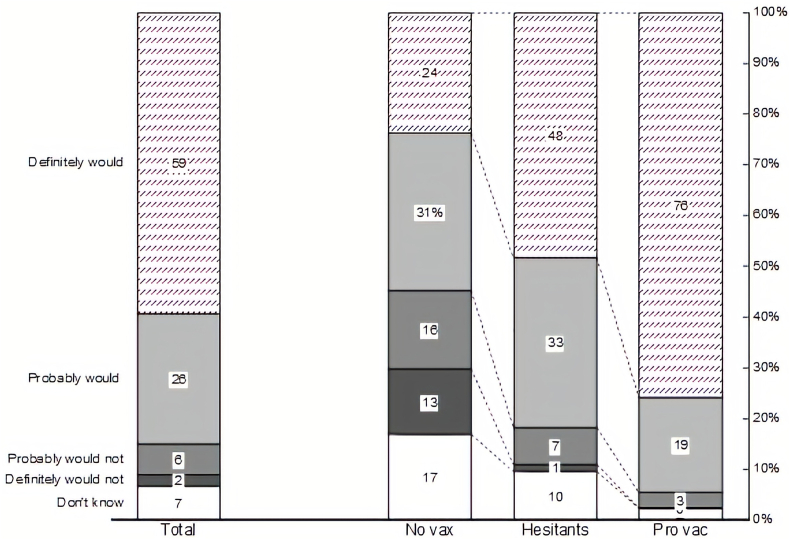
COVID-19 vaccine acceptance and general vaccine attitudes. *Notes*: The figure shows responses to the question: “If a vaccine against COVID-19 became available for everyone tomorrow, do you think you would or would not get vaccinated?” The bar on the left reports the breakdown for all respondents of the April 2020 survey (N = 1194). The other 3 columns report the breakdown for three categories of respondents: “no vax” (N = 148), “hesitants” (N = 431) and “pro vac” (N = 615). We assign respondents to one of these categories using ther answers to the question on general vaccination attitudes. See Section S.2 in the Supplementary Materials for details on the construction of these categories.

In our nationally representative sample, 85% of respondents were either definitely or probably willing to become vaccinated against COVID-19 (see Fig. 1, left bar). Only 8% said that they would either probably or definitely not take the vaccine. Attitudes towards vaccination in general correlated with willingness to receive a potential COVID-19 vaccine. In the group of people generally favorable to vaccination (i.e. the ProVac group), 95% stated that they would like to be vaccinated. However, even among the most skeptical – who believed that vaccines cause autism and have few demonstrable benefits – 24% would “definitely” like to be vaccinated and another 31% would probably do so. Only 29% thought they would probably or definitely not do so. A similar pattern emerged for the question about making COVID-19 vaccination mandatory, with 36% of the most skeptical respondents favoring a legal obligation to be vaccinated (see Supplementary Fig. S2).

**Fig. 2:**
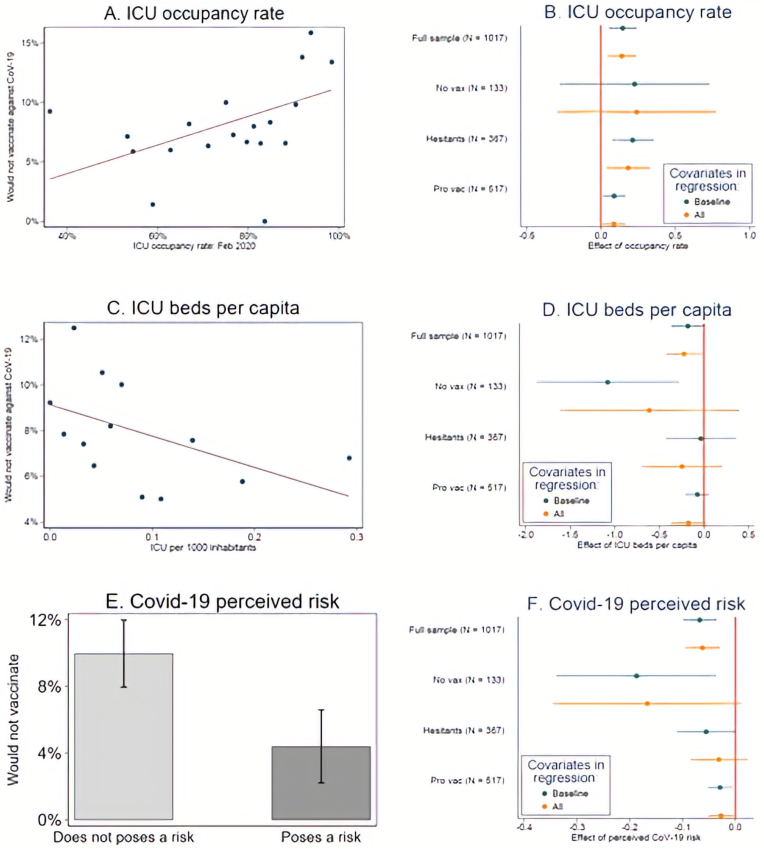
ICU availability, perceived risk and unwillingness to get vaccinated against COVID-19. *Notes*: Resistance to vaccinate against COVID-19 is from the question: “If a vaccine against COVID-19 became available for everyone tomorrow, do you think you would or would not get vaccinated?” Respondents who would *not* vaccinate “definitely” and “probably” are coded as resistant. Panel A: unconditional binscatter of February 2020 ICU beds occupancy rate (x-axis) and resistance to COVID-19 vaccine (y-axis). From the full sample of respondents living in England we create 20 bins of roughly equal sample size; the last 2 bins have no variation in occupancy rate (100%) and are combined into a single data point. Panel C: unconditional binscatter of February 2020 ICU beds per 1000 people (x-axis) and resistance to COVID-19 vaccine (y-axis). From the full sample of respondents living in England we create 20 bins of roughly equal sample size; some 30% of respondents live in a local authority without a NHS Trust: these bins are combined into a single data point. Panel E: share of respondents showing resistance to COVID-19 vaccine among those who state that COVID-19 does *not* poses a major risk to anyone in the household (left bar) and those who state that it does (right bar). The whiskers show the standard errors of the estimates. Panel B, D and F: OLS estimates and 95% confidence intervals from. COVID-19 Vax Resistance_i_ = β_0_ + β_1_ OR_i_ + β_2_ ICU_i_ + β_3_ CoV19 Risk_i_ + β_X_ X_i_ + u_i_ Where *COVID-19 Vax Resistance* = 1 if respondent states that he would “definitely” or “probably” not vaccinate against COVID-19, and the other variables are defined in the footnote of Table 2. Panel B: estimates of *β*_*1*_. Panel D: estimates of *β*_*2*_. Panel F: estimates of *β*_*3*_. The specification with baseline covariates includes an indicator for whether the respondent knows someone infected with COVID-19. The specification with all covariates includes all explanatory variables in col. 4 of Table 2. “Full sample” includes all respondents living in England. The other three samples report estimates from three regressions estimated on the three samples: “no vax,” “hesitants,” and “pro vac.” Respondents are assigned to one of these categories using their answers to a question on general vaccination attitudes. See Section S.2 in the Supplementary Materials for details on the construction of these categories. Standard errors are clustered at the level of the local authority (269 clusters).

We observed that lower availability of public health resources was associated with lower willingness to become vaccinated during the recent COVID-19 crisis (Fig. 2). In particular, in the April 2020 cross-section, lower availability of ICU units in an area and a higher occupancy ratio of these ICUs were both associated with more limited support for COVID-19 vaccination, while late-February 2020 ICU capacity use was strongly correlated with COVID-19 mortality rates at the peak of the crisis (Fig. S4 and Table S4 in the Supplementary Material).

In particular, the highest share of respondents unwilling to be vaccinated lived in areas where ICU units were near capacity by the end of February 2020, immediately before the COVID-19 crisis hit the UK (see Panel A of Fig. 2 which displays a simple binscatter of willingness to vaccinate against ICU occupancy in late February 2020; for expositional clarity, we averaged values of observations into equal-sized bins to produce this and the following graph). The effect was independent of pre-existing attitudes towards vaccination – both sceptics and vaccination supporters showed higher rates of support for COVID-19 -vaccination where England's National Health Service had enough free ICU capacities.

This positive association between ICU occupancy and vaccination scepticism also holds in a multivariate setting: Fig. 2, Panel B plots the regression coefficients for two specifications – a baseline adjusting for perceived risk, the other main explanatory variable (either ICU occupancy or ICU per capita availability), plus an indicator variable for knowing someone with COVID-19, and one using a full set of covariates including age, socio-economic status, education, gender, marital status, and regional fixed effects. In both cases, the effect of ICU occupancy was highly significant. As the disaggregation by subgroup showed, the biggest effect was visible among vaccination hesitants – those with a somewhat ambivalent attitude towards vaccines. There was also a significant but much smaller effect for the pro vaccination group. The no vaccination group on average appeared to react to ICU occupancy, but estimates were not significantly different from zero.

A similar pattern was apparent for another indicator of public health system capacity – ICU beds per 1000 inhabitants (Fig. 2, Panels C and D). Where the NHS had ample hospital beds available before the COVID-19 crisis hit, vaccination unwillingness in general was much lower. The lower the number of ICU units in an area, the more people indicated that they would be unwilling to be vaccinated. The effect was significant at the 90% level without covariates and 95% level with covariates. The average coefficient was largest for vaccination sceptics, but with large standard errors. Hesitants appeared less influenced by ICU provision.

Neither ICU provision per capita nor occupancy rates in October 2019 predicted attitudes towards COVID-19 vaccination, nor did they have predictive power for trust in medical experts and scientists during the COVID-19 crisis (see Table S3) – it was only health care availability during the coronavirus outbreak (i.e. ICU availability in February 2020) that mattered for attitudes.

The ICU availability immediately prior to the COVID outbreak was also a significant predictor of trust in science and the medical profession: where the NHS ran out of ICU capacity during the COVID-19 crisis, trust in science and medical experts was markedly lower (see Table 2 with OLS estimates and Table S5 with Probit estimates). In areas where 90% or more of ICU beds were occupied by late February 2020, only 35% of respondents had a lot of confidence, compared with 42% in the sample overall. The same pattern was true – in reverse – for ICU provision per capita, with greater numbers of ICU units in the nearest hospital predicting more trust in health experts and scientists.

**Table 2:** ICU beds occupancy rate and trust in health experts and scientists.

	Trust in health experts			
	^1^	^2^	^3^	^4^
Closest ICU: occupancy rate (Feb 2020)	−0.221**	−0.207**	−0.227**	−0.230**
	[0.0931]	[0.0922]	[0.087]	[0.089]
ICU per 1000 people (Feb 2020)	0.328* [0.191]	0.250	0.158	0.207
		[0.195]	[0.203]	[0.224]
COVID-19 poses major risk	0.017	0.022	0.054	0.053
	[0.033]	[0.033]	[0.033]	[0.033]
Knows someone with COVID-19	0.055	0.049	0.027	0.027
	[0.037]	[0.037]	[0.037]	[0.037]
COVID-19 deaths per 1000 people	−0.114	−0.146	−0.199	−0.180 [0.150]
	[0.134]	[0.132]	[0.126]	
Oct 2019 vaccination attitudes	No	Yes	Yes	Yes
Demographic controls	No	No	Yes	Yes
Local Authority characteristics	No	No	No	Yes
*R*^*2*^	0.011	0.032	0.071	0.072
Mean dep var	0.418	0.418	0.418	0.418
Observations	1017	1017	1017	1017

*Notes*: The table reports OLS estimates of the following regression.

Trust_i_ = β_0_ + β_1_ OR_i_ + β_2_ ICU_i_ + β_3_ COVID-19 Risk_i_ + β_4_ COVID-19 Exposure+ β_5_ COVID-19 Deaths_i_ + β_X_ X_i_ + u_i_

Where *Trust* is an indicator variable = 1 if respondent reports “a great deal of trust” in health experts and scientists, *OR* is the occupancy rate of ICU beds in the NHS trust that is closest to the zip code where the respondent lives, *ICU* is the number of ICU beds per 1000 people in the local authority where the respondent lives, *COVID-19 Risk* is an indicator variable = 1 if the respondent states that COVID-19 poses a major risk to either himself or someone living in his household, *COVID-19 Exposure* is an indicator variable = 1 if respondent knows someone infected with COVID-19 and *COVID-19 Deaths* is the number of COVID-19 deaths per 1000 people in the local authority as of 10 April 2020. Col. 1 includes only these covariates. Col. 2 includes the answers to 3 questions on vaccination attitudes asked in October 2019: “should unvaccinated kids be allowed to attend school?” “should parents who choose not to vaccinate their kids be banned from childcare benefits?” and “should parents who choose not to vaccinate their kids be fined?“. For each of these questions, we create an indicator variable = 1 if the respondent stated that he would punish parents who choose not to vaccinate their kids, showing support for measures promoting vaccination. Col. 3 adds a gender indicator variable, 3 age groups dummies (18–34; 35–54 and 55+), an indicator variable for high social status (level A-C1 in the NRS classification) and 3 education dummies (low, mid, and high level). Col. 4 adds characteristics of the local authority where the individual lives: the share of people above 65 years old and the life expectancy at 65 for both men and women. The sample includes all respondents re-contacted in April 2020 and living in England. Standard errors are clustered at the level of the local authority (269 clusters).

Personal risk was also an important determinant of attitudes: survey respondents who thought that COVID-19 posed a clear risk to themselves or family members were much more likely to be willing to be vaccinated (see Fig. 2, Panels E and F). The share of respondents unwilling to be vaccinated in the at-risk group was a mere 4%, compared with 10% in the no-risk group. The difference was largest among vaccination sceptics. Hesitants and pro vaccination respondents also reacted to personal risk, but the effects were smaller; for the hesitants, the effect became insignificant when adjusting for the full set of covariates.

Finally, we examined whether the COVID-19 crisis changed vaccination attitudes at the individual level over time. As individuals were asked the same questions about support for punishing vaccination evaders twice – in October 2019 and April 2020 – we could directly examine this issue. Of the three measures – keeping non-vaccinated children out of school, cutting parents’ benefits, and fining the parents if a child was not vaccinated – only one measure (fining) saw a major increase in support during the COVID-19 crisis (Fig. S3). No additional support for withholding attendance or cutting benefits was forthcoming in the sample. At the same time, 1 out of 10 respondents who stated that parents who refused to vaccinate should not be fined changed their mind by April 2020, a mere 7 months later. The rise in support for fines was mainly driven by those with pro-vaccination attitudes – and to a lesser extent, the vaccination hesitants. Respondents with anti-vaccine beliefs did not change their support for penalties, despite the severity of the COVID-19 crisis and their frequent willingness to become vaccinated against COVID-19.

## Discussion

Can vaccination stop COVID-19? Given the new virus’ infectiousness, for vaccination to succeed, take-up rates will have to be high. High support cannot be taken for granted, given the general rise of vaccine hesitancy in recent years.

Our nationally representative survey of UK respondents showed substantial support for vaccination across all socio-economic groups, and even among vaccination sceptics. Some of this reflects perceived personal risk: Having a close family member at risk sharply increased support for vaccination (Fig. 2). Even respondents who believed that vaccines can cause autism, have other severe side-effects, generate few benefits, and are mainly prescribed because of financial interests of the pharmaceutical industry, were overwhelmingly willing to become vaccinated against COVID-19 in April 2020 (Fig. 1). This implies that vaccine hesitancy is unlikely to impede herd immunity against COVID-19 through vaccination.

Areas with more limited ICU capacity experienced sharply higher case fatality rates. ICU availability was a major concern among the public during the pandemic's peak, in March and April 2020. We found that in areas where ICU availability was limited for exogenous reasons, vaccination support is markedly lower – and so is trust in medical experts and scientists.

In general, vaccination can substitute for health interventions post-infection – higher immunization rates reduce the need for post-infection treatment. Hence, vaccination willingness should in principle be inversely related to the availability of health care support. In contrast, we find lower availability of public health resources to be associated with sharply lower willingness to become vaccinated during the recent COVID-19 crisis. As discussed above, in the April 2020 cross-section, lower availability of ICUs in an area and a higher occupancy ratio were both associated with more limited support for COVID-19 vaccination.

We used the occupancy rate in the nearest NHS hospital immediately before the crisis (late February 2020) as an explanatory variable,^24^ for three reasons. First, it is an important predictor of mortality rates: As discussed above, late February ICU capacity use was strongly correlated with COVID-19 mortality rates at the peak of the crisis. Second, late February ICU occupancy was excludable from the severity of the COVID-19 shock, almost no hospitalizations had occurred by then. Late February occupancy is therefore a plausible shifter of hospital capacity, reflecting the seriousness of other demands on the local health system. Third, ICU capacity was a major concern among the public during the COVID-19 crisis, as evidenced by Google search term frequency: Fig. S1 in the Appendix shows how search frequency on Google peaked in late March/early April – the time of our follow-up survey.

Our results underline the importance of trust and confidence in mainstream medicine as pre-condition for positive attitudes towards vaccination, as previously reported.^12^ It also highlights the risk of a vicious circle, where negative attitudes towards vaccination lead to lower vaccination rates, greater numbers of severely ill patients in times of crisis, more pressure on ICUs, and in turn, less trust in the medical profession and science in general. This finding in particular highlights an important synergy between public health system performance and the public's support for public health measures like large-scale vaccination.

In conclusion, our results suggest that the availability of sufficient ICU places can contribute to building trust in medical experts and boosts the willingness to become vaccinated against COVID-19.

## Contributorship statement

All four authors have contributed equally to all aspects of the article (planning, conducting, and reporting the work) and are joint first authors (and listed in alphabetical order). They all affirm that the manuscript is an honest, accurate, and transparent account of the study being reported; that no important aspects of the study have been omitted; and that any discrepancies from the study plan have been explained.

## Declaration of competing interest

The authors declare that they have no known competing financial interests or personal relationships that could have appeared to influence the work reported in this paper.
