# Supplementary material for: Impact of COVID-19 and intensive care unit capacity on vaccination support: Evidence from a two-leg representative survey in the United Kingdom
Source: J Virus Erad. 2021 May 17;7(2):100044. doi: 10.1016/j.jve.2021.100044 (PMC8127519; doi:10.1016/j.jve.2021.100044)
Supplement: Multimedia component 1 [file mmc1.docx]

ONLINE-ONLY SUPPLEMENTARY MATERIAL

S.1 Data

*Surveys*. Survey data comes from two surveys conducted on 6-7 October 2019 and on 9-16 April 2020. Section S.3 of the Supplementary Material reports the questions used in this study. The surveys were administered online by the company YouGov and consisted in October 2019 of a sample of 1653 respondents representative of the population living in England, Scotland and Wales. All of the original 1653 respondents of October 2019 were re-contacted in April 2020, and 1194 (72%) participated to the follow-up survey. The characteristics of survey respondents in the two waves (Table 1, col. 3-4) were broadly representative of the general population of Great Britain (Table 1, col. 1-2). Attrition was balanced across all characteristics but one (age) (Table 1, col. 5-6).

*NHS variables*. ICU occupancy rate and ICU beds per 1000 people were calculated from the NHS series “Critical Care Bed Capacity and Urgent Operations Cancelled 2019-20,” maintained by the NHS.^[[1]](#footnote-1)^ The February 2020 series were used in the current study. Occupancy rate is ICU beds occupied on the last Thursday of February 2020 as a share of total ICU beds available on the same date. This number was computed for each of the NHS Trusts in England and its address was geolocated. The 3-digits zip-code of survey respondents was then used to find the NHS Trust that is closest to their residence. ICU beds per 1000 people was calculated combining NHS data on ICU beds available with 2018 population estimates at the local authority level (from ONS).^[[2]](#footnote-2)^ Every NHS Trust was first assigned to the local authority where its address is located. Next, ICU beds were aggregated at the level of the local authority and then divided by people (in thousands). Finally, ICU beds per 1000 people were assigned to every respondent based on the local authority where her/his 3-digits zip-code is located.

*Other covariates.* In some specifications were included the share of people older than 65 years and life expectancy at 65 for both men and women. These variables were observed at the local authority level and refer to 2018. They were produced by ONS.^[[3]](#footnote-3)^ These variables were assigned to every respondent based on the local authority where her/his 3-digits zip-code is located.

S.2 Construction of the categories No Vac, Hesitants, Pro Vac

Respondents were assigned to one of three categories of vaccination attitudes: “no vax,” “hesitants,” and “pro vac” based on their answers to the following question:

*For each of the following, please say how true or false you think they are:*

*1. Vaccines have severe adverse effects that are not widely publicized*

*2. Vaccines cause autism*

*3. Vaccines have no clear benefits, as most diseases are not very common and dangerous anymore*

*4. Doctors advise vaccination because they receive money from the pharmaceutical lobby*

*5. Children get more shots than are good for them*

*6. It is better for children to develop immunity by getting sick than by getting a shot*

*7. It is better for children to get fewer vaccines at the same time*

*8. Many of the illnesses that shots prevent are severe*

Each question asked the respondent to rate the statement on a 4-levels scale: “definitely true,” “probably true,” “probably false” and “definitely false.” Questions 1-7: “definitely true” answers was coded as -2, “probably true” as -1, “probably false” as +1, and “definitely false” as +2. Question 8: The coding as inverted and -2 points given to a “definitely false,” -1 to “probably false,” +1 to “probably true” and +2 to “definitely true.” All questions: “don’t know” got a 0. The scores from the 8 questions were summed up to compute an aggregate measure of vaccination attitudes. The score ranges from -16 to +16, with lower values associated with greater mistrust in vaccines. Respondents scoring negative values were classified as “no vax,” respondents scoring above 8 as “pro vac,” the rest as “hesitants.”S.3 Appendix Figures and Tables

**Figure S1: Google search term frequencies**

“ICU+Covid”
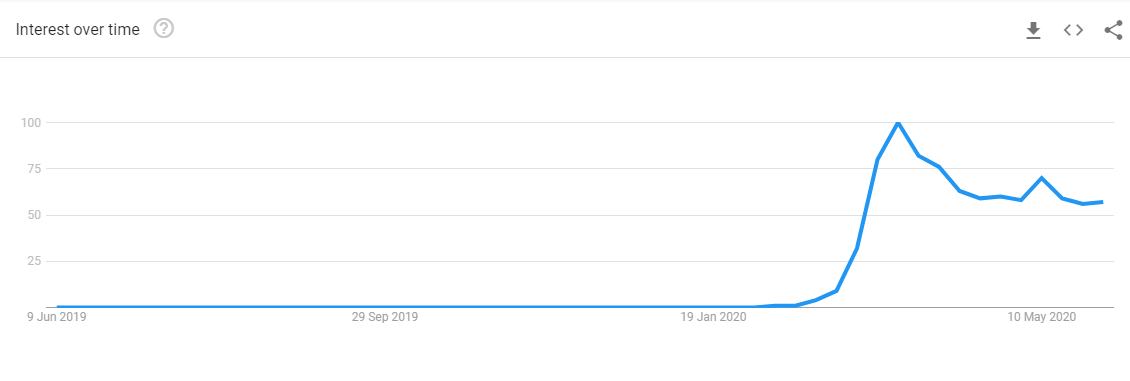


“ICU”
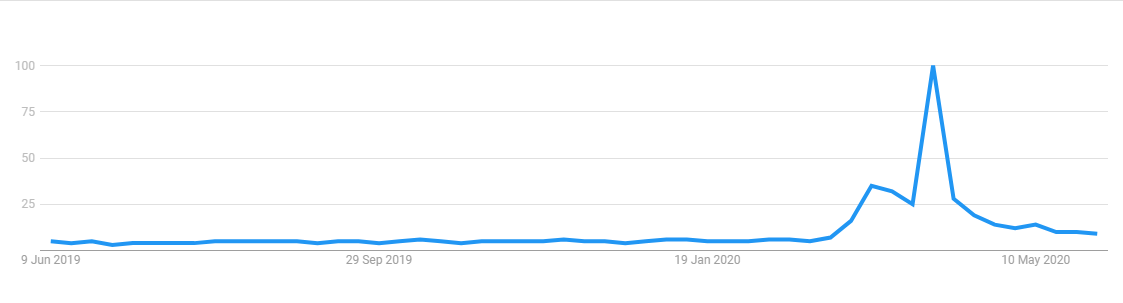


“Intensive care unit”
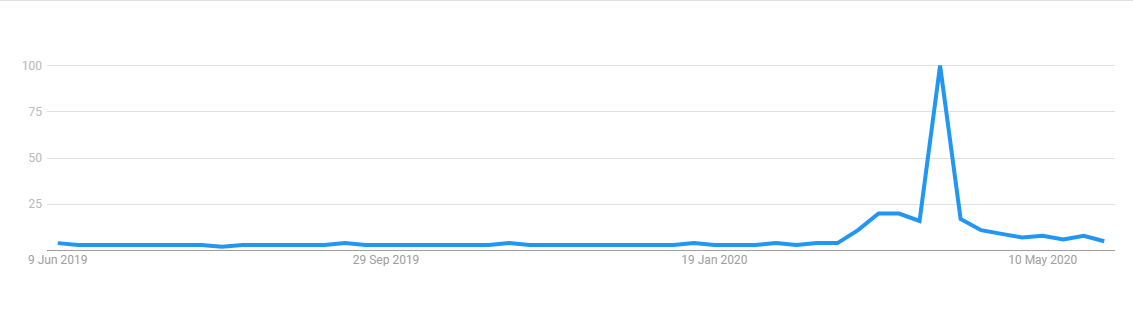


*Notes*: The figure shows the search frequency for three search terms/ combinations: “ICU + Covid” (top panel), “ICU” (middle panel), and “intensive care unit” (bottom panel) in the United Kingdom. In each case, the search term frequency is normalized to 100 for the week of peak search activity. The peak for the latter two terms was during the week of April 5-11, the week of our follow-up survey; for the first panel, it was the week of 22-28 March (results accessed on 8-6-2020).

**Figure S2: Support for mandatory COVID-19 vaccination**

*Notes*: The figure shows the breakdown of responses to the question: “If a vaccine against COVID-19 became available for everyone tomorrow, do you think the UK government should, or should not, make it mandatory for everyone in the UK to be vaccinated?” The bar on the left reports the breakdown for all respondents of the April 2020 survey (N=1194). The other 3 columns report the breakdown for three categories of respondents: “no vax” (N=148), “hesitants” (N=431) and “pro vac” (N=615). We assign respondents to one of these categories using her answers to the questions on general vaccination attitudes. See Section S.2 in the Supplementary Materials for details on the construction of these categories.

**Figure S3: Evolution of general vaccination attitudes**


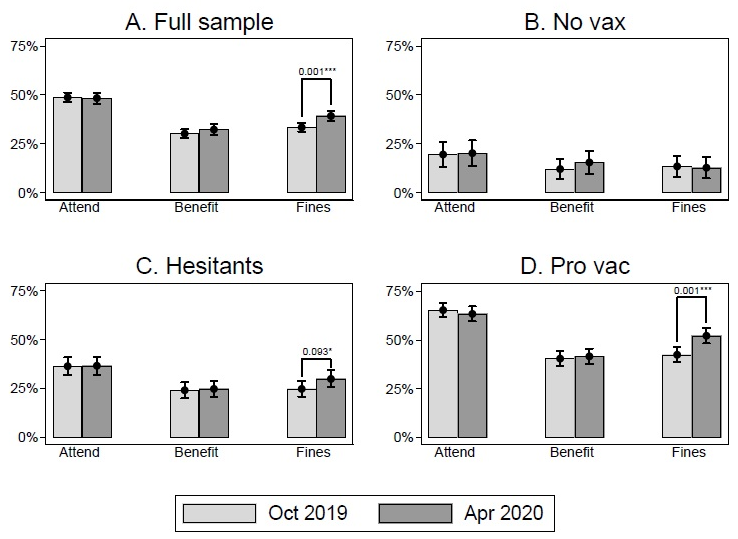


*Notes*: Responses to the three questions on vaccination attitudes asked on October 2019 and again on April 2020: “should unvaccinated kids be allowed to attend school?” (label: “Attend”); “should parents who choose not to vaccinate their kids be banned from childcare benefits?” (label: “Benefit”); and “should parents who choose not to vaccinate their kids be fined?” (label: “Fines”). For each of these questions, an indicator variable was created which was 1 if the respondent stated that he would punish parents who choose not to vaccinate their kids, showing support for measures promoting vaccination: the height of the bars shows the share of people who stated that they would punish these parents, the whiskers around the bars indicate the standard errors of these estimates. Point estimates that are significantly different from each other at a level of 10% or less are indicated explicitly, with *p*-value reported on top. McNemar test on paired data yields similar (but stronger) results: only the proportion of support for fines grows significantly, and *p*-values are <0.0000 (full sample and pro vac) and 0.0376 (hesitants). Panel A: results for all respondents of the two surveys (N= 1653 in October 2019 and N=1194 in April 2020). Panel B: results for respondents classified as “no vax” (N=148). Panel C: results for respondents classified as “hesitants” (N=431). Panel D: results for respondents classified as “pro vac” (N=615). We assign respondents to one of these categories using her answers to the question 4 in the questionnaire on general vaccination attitudes asked in April 2020. See Section S.2 in the Supplementary Materials for details on the construction of these categories.

**Figure S4**. Determinants of COVID-19 lethality.

| 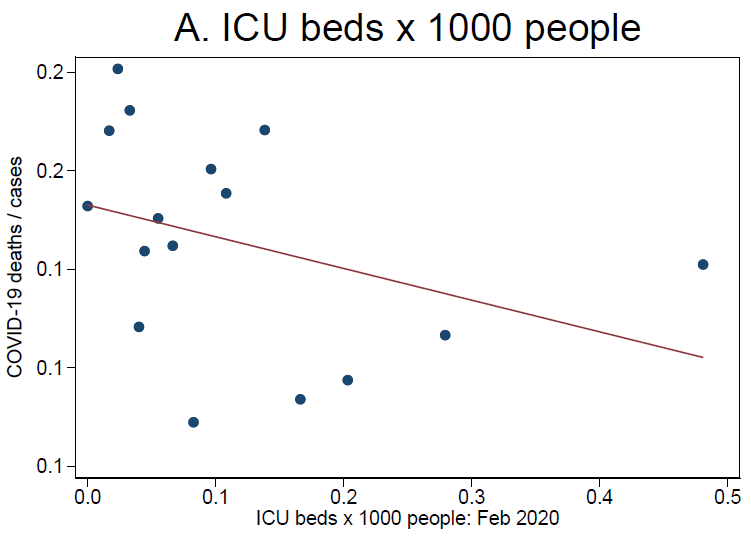 | 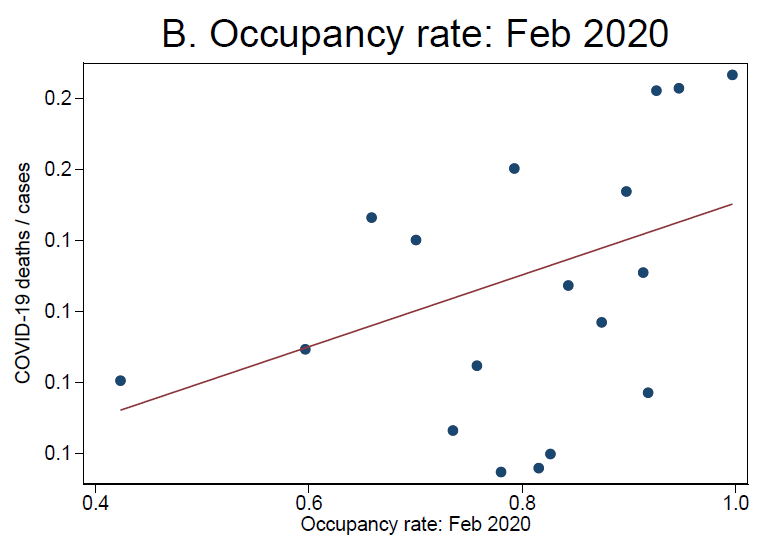 | 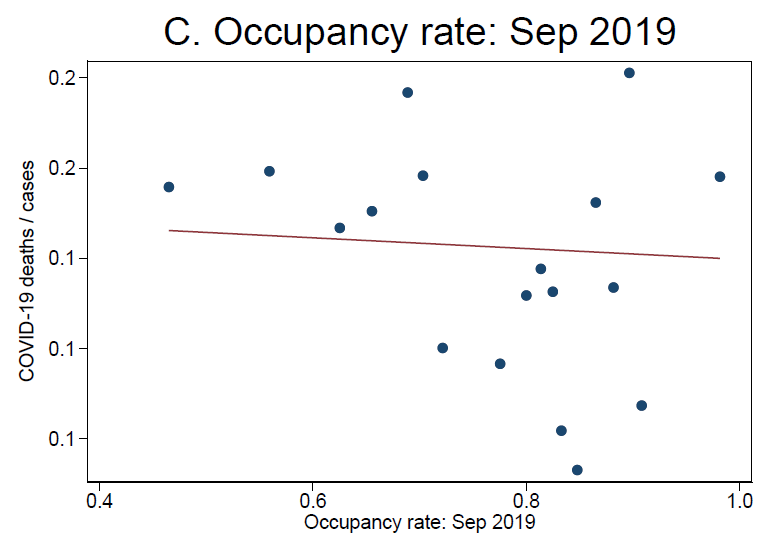 |
| --- | --- | --- |

*Notes*: Determinants of COVID-19 lethality (COVID-19 deaths per cases). Panel A: unconditional binscatter of February 2020 ICU beds per 1000 people (x-axis) and COVID-19 lethality (y-axis). From the full sample of local authorities for which we have information of COVID-19 cases we create 20 bins of roughly equal sample size. Panel B: unconditional binscatter of February 2020 ICU occupancy rate (x-axis) and COVID-19 lethality (y-axis); the last 3 bins have no variation in occupancy rate (100%) and are combined into a single data point. Panel C: unconditional binscatter of September 2019 ICU occupancy rate (x-axis) and COVID-19 lethality (y-axis); the last 2 bins have no variation in occupancy rate (100%) and are combined into a single data point.

**Table S1**. Determinants of response rate in April 2020: no selection bias.


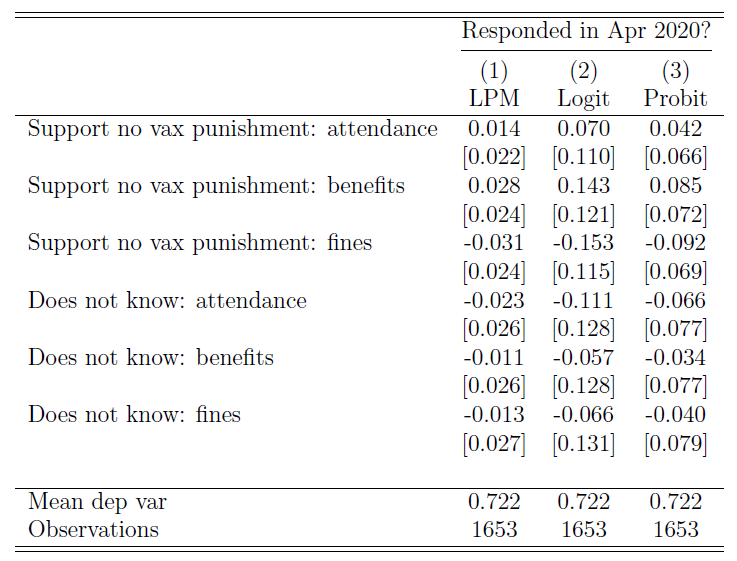


*Notes*: The table reports estimates of bivariate regressions where the dependent variable is an indicator variable = 1 if the individual was successfully re-contacted in April 2020. Dependent variables are indicator variables =1 if the individual stated in October 2019 that she supported penalties for no vax parents (rows 1-3) and indicator variables =1 if the individual replied “don’t know” to the same questions (rows 4-6). Estimation method is: OLS (col. 1), logit (col. 2) and probit (col. 3). Sample includes all respondents to the October 2019 survey. Robust standard errors in parentheses.

**Table S2**. Determinants of attitudes towards COVID-19 vaccination: probit estimates.

**
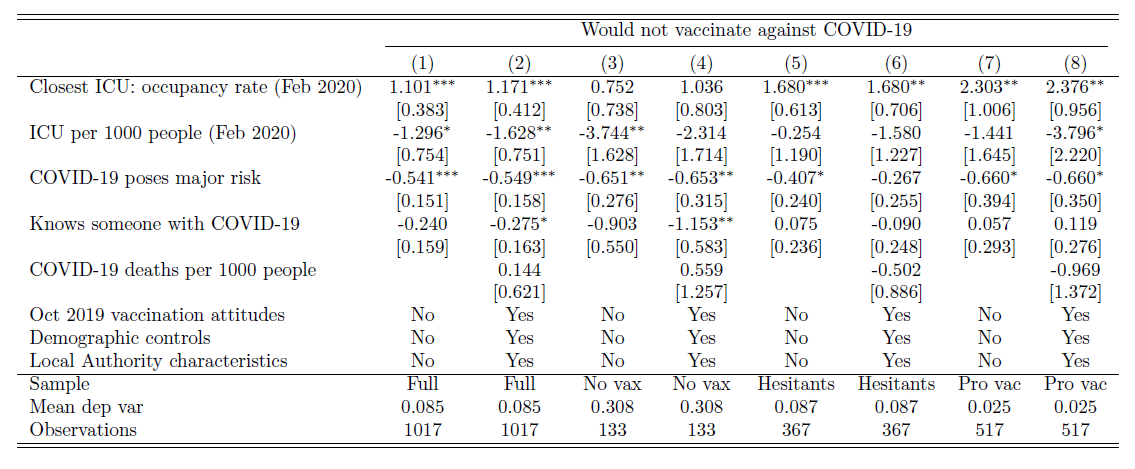
**

*Notes*: The table reports probit estimates of the following regression:

*COVID-19 Vax Resistance_i_ = β_0_ + β_1_ OR_i_ + β_2_ ICU_i_ + β_3_ CoV19 Risk_i_ + β_X_ X_i_ + u_i_*

where *COVID-19 Vax Resistance* = 1 if respondent states that he would “definitely” or “probably” not vaccinate against COVID-19, and the other variables are defined in the footnote of Table 2. Col. 1, 3, 5 and 7: the specification includes an indicator for whether the respondent knows someone infected with COVID-19. Col. 2, 4, 6 and 8: the specification includes all explanatory variables in col. 4 of Table 2. “Full sample” includes all respondents living in England. The other three samples report estimates from regressions estimated on the three samples: “no vax,” “hesitants,” and “pro vac.” Respondents are assigned to one of these categories using their answers to a question on general vaccination attitudes. See Section S.2 in the Supplementary Materials for details on the construction of these categories. Standard errors are clustered at the level of the local authority (269 clusters).

**Table S3**. Determinants of attitudes towards COVID-19 vaccination and trust in experts: no effect of past ICU capacity.


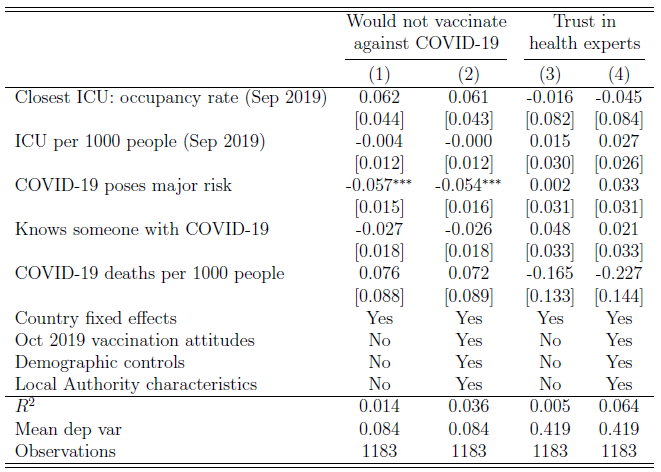


*Notes*: The table reports OLS estimates of the following regression:

*Y_i_ = β_0_ + β_1_ OR_i_ + β_2_ ICU_i_ + β_3_ COVID-19 Risk_i_ + β_4_ COVID-19 Exposure+ β_5_ COVID-19 Deaths_i_ + β_X_ X_i_ + u_i_*

where the dependent variable *Y* is: col. 1-2: an indicator variable = 1 if respondent would “definitely” or “probably” not vaccinate against COVID-19; col. 3-4: an indicator variable = 1 if respondent reports “a great deal of trust” in health experts and scientists. *OR* in September 2019 is the occupancy rate of ICU beds in the NHS trust that is closest to the zip code where the respondent lives, *ICU* is the number of ICU beds per 1000 people in September 2019 in the local authority where the respondent lives, *COVID-19 Risk* is an indicator variable = 1 if the respondent states that COVID-19 poses a major risk to either himself or someone living in his household, *COVID-19 Exposure* is an indicator variable = 1 if respondent knows someone infected with COVID-19 and *COVID-19 Deaths* is the number of COVID-19 deaths per 1000 people in the local authority as of 10 April 2020. Col. 1 and 3 include only these covariates. Col. 2 and 4 also include: the answers to 3 questions on vaccination attitudes asked in October 2019: “should unvaccinated kids be allowed to attend school?” “should parents who choose not to vaccinate their kids be banned from childcare benefits?” and “should parents who choose not to vaccinate their kids be fined?”. For each of these questions, we create an indicator variable = 1 if the respondent stated that he would punish parents who choose not to vaccinate their kids, showing support for measures promoting vaccination. Col. 2 and 4 also include a gender indicator variable, 3 age groups dummies (18-34; 35-54 and 55+), an indicator variable for high social status (level A-C1 in the NRS classification) and 3 education dummies (low, mid, and high level), the share of people above 65 years old and the life expectancy at 65 for both men and women. The sample includes all respondents re-contacted in April 2020. Standard errors are clustered at the level of the local authority (302 clusters).

**Table S4**. Determinants of COVID-19 lethality.


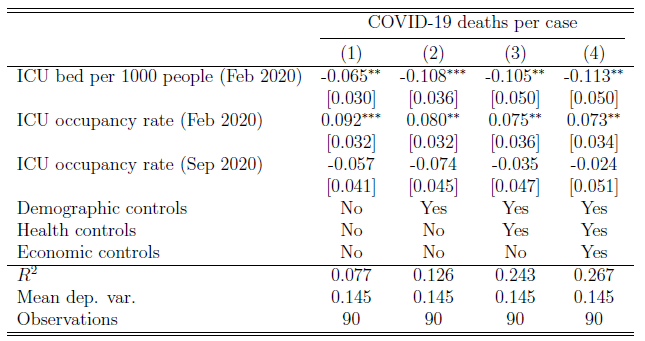


*Notes*: The table reports OLS estimates of the following regression:

*COVID-19 Lethality_i_ = β_0_ + β_1_ ICU Feb 2020_i_ + β_2_ OR Feb 2020_i_ + β_3_ OR Sep 2019_i_ + β_X_ X_i_ + u_i_*

where the unit of observation are English local authorities, and *COVID-19 Lethality* is number of COVID-19 deaths (as of 10 of April 2020) per number of COVID-19 cases (as of 16 April 2020). *ICU Feb 2020* is the number of ICU beds available on February 2020 per 1000 people, *OR Feb 2020* and *OR Sep 2019* are the occupancy rate of ICU beds in February 2020 and September 2019 respectively. Col. 1 includes only these covariates. Col. 2 adds demographic characteristics: the share of population above 65 years old, log density and log male-female ratio. Col. 3 adds covariates for the general health of the population: life expectancy at 65 for men and for women, share of people with “a lot” and “a little” limitation in day-to-day activities due to health issues, share of people with “fair,” “bad” and “very bad” health (omitted categories: “good” and “very good”), and share of people providing non-paid care. Col. 4 include two economic covariates: the share of people owning their home outright and with a mortgage. The sample includes all local authorities reporting COVID-19 cases as of 16 April 2020 and have at least one NHS trust.

**Table S5**. ICU beds occupancy rate and trust in health experts and scientists: probit estimates.


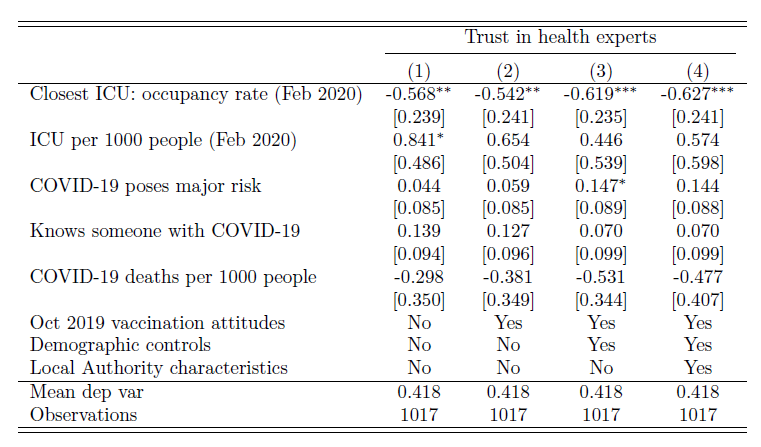


*Notes*: The table reports probit estimates of the following regression:

*Trust_i_ = β_0_ + β_1_ OR_i_ + β_2_ ICU_i_ + β_3_ COVID-19 Risk_i_ + β_4_ COVID-19 Exposure+ β_5_ COVID-19 Deaths_i_ + β_X_ X_i_ + u_i_*

Where *Trust* is an indicator variable = 1 if respondent reports “a great deal of trust” in health experts and scientists, *OR* is the occupancy rate of ICU beds in the NHS trust that is closest to the zip code where the respondent lives, *ICU* is the number of ICU beds per 1000 people in the local authority where the respondent lives, *COVID-19 Risk* is an indicator variable = 1 if the respondent states that COVID-19 poses a major risk to either himself or someone living in his household, *COVID-19 Exposure* is an indicator variable = 1 if respondent knows someone infected with COVID-19 and *COVID-19 Deaths* is the number of COVID-19 deaths per 1000 people in the local authority as of 10 April 2020. Col. 1 includes only these covariates. Col. 2 includes the answers to 3 questions on vaccination attitudes asked in October 2019: “should unvaccinated kids be allowed to attend school?” “should parents who choose not to vaccinate their kids be banned from childcare benefits?” and “should parents who choose not to vaccinate their kids be fined?”. For each of these questions, we create an indicator variable = 1 if the respondent stated that he would punish parents who choose not to vaccinate their kids, showing support for measures promoting vaccination. Col. 3 adds a gender indicator variable, 3 age groups dummies (18-34; 35-54 and 55+), an indicator variable for high social status (level A-C1 in the NRS classification) and 3 education dummies (low, mid, and high level). Col. 4 adds characteristics of the local authority where the individual lives: the share of people above 65 years old and the life expectancy at 65 for both men and women. The sample includes all respondents re-contacted in April 2020 and living in England. Standard errors are clustered at the level of the local authority (269 clusters).

S.4 Survey Questionnaire

Below are listed the survey questions used for the current study. Further survey questions asked referred to a broad set of topics, including UK politics (available on request to the corresponding author).

Questions asked both in October 2019 and April 2020

*Vaccination attitudes*

Q1. Do you think children whose parents have intentionally not vaccinated them should or should not be allowed to attend state schools in the UK?

Q2. Do you think parents who intentionally choose not to vaccinate their children should or should not be banned from receiving childcare benefits?

Q3. Do you think parents who intentionally choose not to vaccinate their children should or should not be fined?

Questions asked in April 2020 only:

Q4. For each of the following, please say how true or false you think they are.

- Vaccines have severe adverse effect that are not widely publicized

- Vaccines cause autism

- Vaccines have no clear benefits, as most diseases are not very common and dangerous anymore

- Doctors advise vaccination because they receive money from the pharma lobby

- Children get more shots than are good for them

- It is better for children to develop immunity by getting sick than by getting a shot

- It is better for children to get fewer vaccines at the same time

- Many of the illnesses that shots prevent are severe

*COVID-19 exposure*

Q5. How many people, if any, do you know personally who have either tested positively or have been in direct contact (e.g. partner, co-worker) with someone who tested positively for COVID-19? (If you have tested positive please include yourself in this number)?

Q6. Do you or some household member suffer from a chronic or long-term disease?

- Yes, I or someone in my household suffer from a chronic or long-term disease

- No, neither I nor someone in my household suffer from a chronic or long-term disease

- Prefer not to answer

*COVID-19 vaccine (attitudes)*

Q7. If a vaccine against COVID-19 became available for everyone tomorrow, do you think you would or would not get vaccinated?

Q8. If a vaccine against COVID-19 became available for everyone tomorrow, do you think the UK government should, or should not, make it mandatory for everyone in the UK to be vaccinated?

Q9. How much do you trust health experts and scientists?

1. Accessed on 29 April 2020 at https://www.england.nhs.uk/statistics/statistical-work-areas/critical-care-capacity/critical-care-bed-capacity-and-urgent-operations-cancelled-2019-20-data/. [↑](#footnote-ref-1)
2. Accessed on 15 April 2020 at <https://www.ons.gov.uk/peoplepopulationandcommunity/populationandmigration/populationestimates/datasets/populationestimatesforukenglandandwalesscotlandandnorthernireland> [↑](#footnote-ref-2)
3. Accessed on 29 April 2020 at: <https://www.ons.gov.uk/peoplepopulationandcommunity/healthandsocialcare/healthandlifeexpectancies/datasets/lifeexpectancyatbirthandatage65bylocalareasuk> [↑](#footnote-ref-3)
